# MAP Kinases and Prostate Cancer

**DOI:** 10.1155/2012/169170

**Published:** 2011-10-20

**Authors:** Gonzalo Rodríguez-Berriguete, Benito Fraile, Pilar Martínez-Onsurbe, Gabriel Olmedilla, Ricardo Paniagua, Mar Royuela

**Affiliations:** ^1^Department of Cell Biology and Genetics, University of Alcalá, Alcalá de Henares, 28871 Madrid, Spain; ^2^Department of Pathology, Príncipe de Asturias Hospital, Alcalá de Henares, 28806 Madrid, Spain

## Abstract

The three major mitogen-activated protein kinases (MAPKs) p38, JNK, and ERK are signal transducers involved in a broad range of cell functions including survival, apoptosis, and cell differentiation. Whereas JNK and p38 have been generally linked to cell death and tumor suppression, ERK plays a prominent role in cell survival and tumor promotion, in response to a broad range of stimuli such as cytokines, growth factors, ultraviolet radiation, hypoxia, or pharmacological compounds. However, there is a growing body of evidence supporting that JNK and p38 also contribute to the development of a number of malignances. In this paper we focus on the involvement of the MAPK pathways in prostate cancer, including the less-known ERK5 pathway, as pro- or antitumor mediators, through their effects on apoptosis, survival, metastatic potential, and androgen-independent growth.

## 1. Introduction

**Figure 1:**
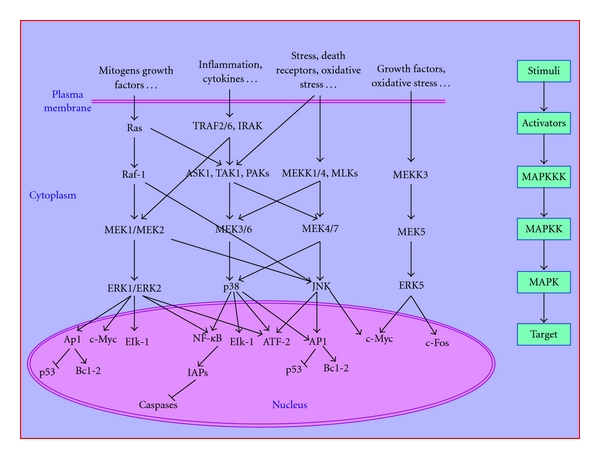
Mitogen-activated protein kinase (MAPK) signaling. MAP kinases are activated by upstream kinases such as MAP kinase kinase (MAPKK), that include MEKs 1, 2, 3, 4, 5, 6, and 7. In turn, MAPKKs are activated by several different MAP kinase kinase kinases (MAPKKKs). Numerous stimulatory factors such as cytokines, mitogens, or death receptors can activate MAPKKKs. Each MAPK, depending on the stimulus and cell type, can phosphorylate different transcription factors.

Mitogen-activated protein kinases (MAPKs) are serine/threonine kinases that mediate intracellular signaling associated with a variety of cellular activities including cell proliferation, differentiation, survival, death, and transformation [1, 2]. The three main members that integrate the MAPK family in mammalian cells are stress-activated protein kinase c-Jun NH2-terminal kinase (JNK), stress-activated protein kinase 2 (SAPK2, p38), and the extracellular signal-regulated protein kinases (ERK1/2, p44/p42) (Figure 1). In addition, other less-characterized MAPK pathways exist, such as the extracellular regulated kinase 5 (ERK5) pathway [3, 4] (Figure 1). Albeit with multiple exceptions, JNK and ERK5 are generally associated with apoptosis induction, while ERK1/2 are generally associated to mitogenesis, and inversely related to apoptosis [3, 4], and contradictory effects on cell death have been described to p38 [5–12].

In mammalian cells, ERK, p38, and JNK activities are, respectively, regulated by different MAPKs cascades, which provide a link between transmembrane signaling and changes in transcription and that are activated in response to different environmental or developmental signals [4] (Figure 1). Depending on the cell type, a particular MAPK cascade may be involved in different cellular responses. The JNK and p38 signaling pathways are activated by proinflammatory (TNF*α*, IL-6 or IL-1) or anti-inflammatory (EGF, TGF-*β*) cytokines, but also in response to cellular stresses such as genotoxic, osmotic, hypoxic, or oxidative stress. The JNK pathway consists of JNK, an MAPKK such as SEK1 (also known as MEK4) or MEK7, and an MAPKKK such as ASK1, MEKK1, mixed-lineage kinase (MLK), or transforming growth factor-*β*-activated kinase 1 (TAK1) [13, 14]. In the p38 signaling pathway, distinct MAPKKs such as MEK3 and MEK6 activate p38, and these can be activated by the same MAPKKKs (such as ASK1 and TAK1) that function in the JNK pathway. In the ERK signaling pathway, ERK1 or ERK2 (ERK1/2) is activated by MEK1/2, which in turn is activated by a Raf isoform such as A-Raf, B-Raf, or Raf-1 (also known as C-Raf) and also by TRAF-2 and TRAF-6. The kinase Raf-1 is activated by the small Ras-like GTPase, whose activation is mediated by the receptor tyrosine kinase (RTK)-Grb2-SOS signaling axis [15]. Members of the Ras family of proteins, including K-Ras, H-Ras, and N-Ras, play a key role in transmission of extracellular signals into cells [16] (Figure 1). 

The aim of this paper was to focus on the possible involvement of MAPKs in several transduction pathways related with prostate cancer development as well as the possible functional role of MAPKs in cell death/survival/proliferation decisions depending on the cell type, stage, and cell stimulus. We also discuss the possible value of members of these pathways as potential therapeutic targets.

## 2. Jun N-Terminal Kinase (JNK)

**Figure 2:**
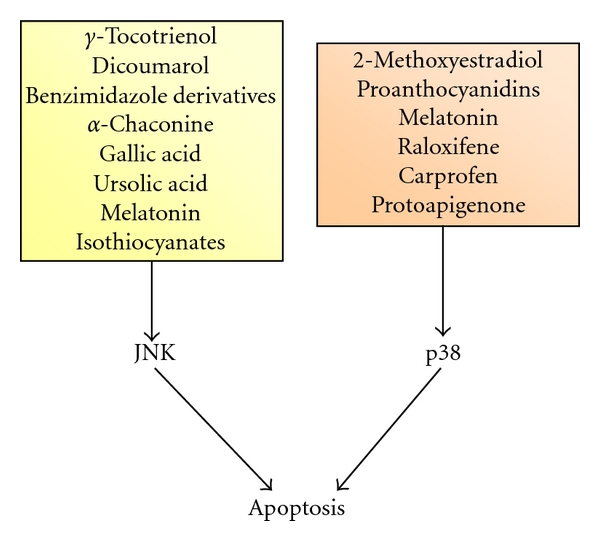
JNK and p38 MAPKs mediate apoptotic cell death induced by a variety of compounds in prostate cancer cells.

JNK proteins, also called stress-activated protein kinases (SAPKs), share a threonine-proline-tyrosine (TPY) motif within their activation loop [17]. They have been involved in development, morphogenesis, and cell differentiation [17]. The earliest discoveries included the identification of the three mammalian JNK genes, namely, JNK1, JNK2, and JNK3 (SAPK-*γ*, SAPK-*α*, and SAPK-*β*, resp.) which can generate 10 isoforms by alternative splicing [18, 19]. Alternative splicing further increases the diversity of JNK proteins; however apart from early biochemical studies on these splice forms [16] their functional significance *in vivo *remains largely unexplored [19]. The products of JNK1 and JNK2 are ubiquitously expressed in almost all cell types and tissues, whereas JNK3 is localized primarily in brain, heart, and testis. Due to their differential expression distribution it is thought that JNK3 presents different functions than JNK1 and JNK2, whereas these latter may have redundant functions [20]. Investigations on JNKs have focused on their activation in response to diverse extracellular stimuli including ultraviolet (UV) and gamma radiation, inflammatory cytokines (IL-6, IL-1, and TNF), and cytotoxic drugs (Figure 2) [21, 22]. These stimuli are able to activate JNK through multiple and even overlapping cascades in which participate members of the small Ras-like GTPases or several MAPKKKs (Figure 1). For its complete activation JNK requires dual phosphorylation of threonine and tyrosine residues. MEK4 and MEK7 preferentially phosphorylate at tyrosine and threonine, respectively [23–27], being both MAPKKs needed to fully activate JNK [4, 28]. Depending on the stimulus and cell type, JNKs phosphorylate different substrates, including transcription factors (AP-1, ATF-2, Elk-1, c-Myc, p53, MLK2) and several members of the Bcl-2 family, among others [17, 20, 29] (Figure 1). 

Several authors suggest that JNK activity is chronically altered in various cancer types such as those of the prostate [30, 31], breast [32, 33], pancreas, or lung [34, 35]. Both JNK1 and 2 have been shown to exert pro- as well as antitumor actions in a number of *in vivo *and *in vitro *models of malignancies [6, 36]. A number of findings suggest that in apoptosis JNKs have opposite functions depending on the cellular stimulus and type or even the JNK isoform. 

Studies into the status of JNK in human prostate tissues are scarce. Both nuclear and total JNK expression seems to be augmented in human malignant prostate epithelium in comparison with normal or benign hyperplasic (BPH) epithelium [30–40]. We are not aware of studies that analyze the activation state of JNK in organ-confined human prostate cancers. Nevertheless in human metastatic lesions, and late-stage carcinomas and metastatic deposits from a murine model of prostate cancer, JNK phosphorylated forms seem to be reduced [39, 41, 42]. 

In spite of its prominent role as a proapoptotic factor, as in other malignances, both pro- and antitumor actions have been attributed to JNK in prostate cancer. Hence, a great number of agents have been shown to trigger apoptosis through JNK. These include gamma-tocotrienol [43], dicoumarol [44], benzimidazole derivatives [45], alpha-chaconine, gallic acid [34], ursolic acid [35], melatonin [36], and isothiocyanates [46, 47] (Figure 2). It is of interest to note that androgen deprivation, the most common therapy used as treatment for advanced prostate cancer, may elicit apoptosis through JNK activation [48]. In the context of its proapoptotic role JNK has been linked to reactive oxygen species (ROS). Some works have highlighted the capability of JNK to trigger apoptosis through ROS production in prostate cancer cells [49, 50]. Conversely, ROS may induce apoptosis acting through JNK activation. For instance, both genipin- and guggulsterone-induced prostate cancer cell apoptoses are caused by ROS-dependent JNK activation [51, 52]. Regarding to its antiapoptotic function, JNKs have been involved in protection from serum starvation-, Fas-, and (at early phase) glucose deprivation-induced apoptosis [53–55]. 

Besides promoting prostate cancer development by protecting cells from apoptosis, JNK may be involved in prostate cancer metastasis, through its ability to regulate cell adhesion, invasion, and migration. Thus, JNK has been shown to promote the expression of some proteins responsible for extracellular matrix degradation during invasion in prostate cancer cells, such as matrix metalloproteinases (MMPs)-2 and -9, and urokinase-type plasminogen activator (u-PA) [56–58]. Moreover, Kwon et al. [56] reported that chemical inhibition of JNK in DU145 prostate cancer cells reduces both cell migration and vascular-endothelial growth factor (VEGF) expression, a proangiogenic factor that may facilitate tumor growth and metastasis.

## 3. Stress-Activated Protein Kinase 2 (p38)

p38 family members contain a TGY (threonine-glycine-tyrosine) motif in their activation loop. These kinases play roles in cell differentiation, growth, proliferation, survival, and apoptosis [59–61]. Four isoforms of p38 exist, namely, p38*α*, *β*, *γ*, and *δ*, which exhibit some different functional properties. Whereas p38*α* and p38*β* are ubiquitously expressed, p38*γ* and p38*δ* expression is restricted to some tissues such as muscle, testis, pancreas, lung, kidney, or endocrine glands [62–64]. p38 is activated in cells in response to stress signals, proinflammatory (TNF*α*, IL-6 or IL-1) or anti-inflammatory (EGF, TGF-*β*) cytokines, UV radiation, and heat and osmotic shock [59, 65]. A great number of MAPKKs and MAPKKKs (e.g., Mlk1-3, MEKK1-4, TAK, ASK1/2) upstream of p38 have been identified. Both MAPKKs and MAPKKKs are generally activated by small Ras-like GTPases as Rac1, Cdc42, RhoA, and RhoB [64]. Activated p38 phosphorylates and regulates many transcription factors (including ATF-2, NF-*κ*B, Elk-1, Max, MEF-2, Mac, p53, or Stat1) [65–67] and other cell cycle and apoptotic mediators (e.g., Cdc25A, Bcl-2) [61]. p38 has been shown to enhance cell survival in response to stress stimuli, for instance, in response to DNA damage [61–68]. Triggering of pro- or antiapoptotic p38-mediated response seems to depend on the stimulus, the cell system, and the p38 isoform involved [64]. 

Several studies suggest that p38 play an important role in leukemia [64], lymphomas [69], and a number of solid malignances such as breast [65], prostate [70], gastric [71], or lung [72] cancers.

Both p38 and its active form p-p38, as well as some upstream kinases (PAK1, MEK6, MEK4), are overexpressed in human cancerous prostatic epithelium [11, 30, 41]. This agrees with the enhanced levels of the phosphorylated form of the well-established p38 substrates Elk-1 and ATF-2 at the same compartment [11]. Uzgare et al. [41], using a transgenic mouse model for prostate cancer, described that p-p38 is overexpressed in prostatic intraepithelial neoplasia (PIN), well-differentiated and moderately differentiated cancers while was reduced or absent in late-stage adenocarcinomas and metastatic deposits. However, like in other tissues, studies focused on p38 function in the prostate malignancy reveal that this MAPK can elicit multiple and even opposite responses, which seem to vary depending on the cell system and context.

A proapoptotic role for p38 has been established in a number of prostate cancer in vitro models and conditions. p38 promotes apoptosis induced by 2-methoxyestradiol [5], melatonin [6], proanthocyanidins [7], raloxifene [8], carprofen [9], or protoapigenone [10] (Figure 2). By contrast, p38 exerts a protective effect in TNF-induced apoptosis in LNCaP cells, which represents a good model of well-differentiated tumor [11].

In spite of having a prominent proapoptotic role p38 may contribute to prostate cancer progression by promoting tumor growth, androgen independence acquisition, and metastasis. It has been proposed that IL-6 may support androgen-independent tumor growth by enhancing androgen receptor (AR) expression/activity. Lin et al. [73] demonstrated that, in turn, the IL-6-induced androgen response depends on p38 activity. p38 seems to play a critical role in hypoxia-reoxygenation-induced increase in AR activity, as well as increased survival, clonogenicity, and invasiveness in prostate cancer cells [74], thus providing additional support for a role for p38 in androgen dependence acquisition. Huang et al. [75] showed in PC3 cells that p38 MAPK is necessary for TGF-*β*-mediated activation of MMP-2 and cell invasion in prostate cancer. Moreover, p38 has been involved in the invasion and migration abilities of the prostate cancer DU145 cells, by enhancing the expression of MMPs-2 and -9, and urokinase-type plasminogen activator (u-PA) [76]. Xu et al. [77] also described MEK4 as a regulator and activator of MMP-2. In agreement, Tang and Lu [78] found that p38 activity contributes to adiponectin-induced integrin expression and migration capability of human prostate cancer cells. Therefore, and in spite of displaying proapoptotic functions, p38 may constitute a target for prostate cancer treatment given its demonstrated contribution to some prostate cancer hallmarks, as androgen dependence and metastatic phenotype acquisition.

## 4. Extracellular Signal-Regulated Protein Kinases (ERK1/2)

ERK has a threonine-glutamic acid-tyrosine (Thr-Glu-Tyr) motif [79, 80] that plays a central role in stimulation of cell proliferation [81, 82]. The biological consequences of phosphorylation of ERK substrates include increased proliferation, differentiation, survival [83], angiogenesis [84], motility [85], and invasiveness [86]. The two isoforms of ERK, referred to as ERK1 (or p44) and ERK2 (or p42), share 85% amino acid identity and represent a convergence point for mitogenic signaling from a diverse array of pathways [87–89]. Both are ubiquitously expressed, although their relative abundance in tissues is variable. For example, in many immune cells ERK2 is the predominant species, while in several cells of neuroendocrine origin they may be equally expressed [90]. 

The ERK pathway is triggered mainly by mitogens and cytokines (Figure 1), acting through receptor tyrosine kinases, G-protein-coupled receptors, and nonnuclear activated steroid hormone receptors [4, 65]. Most of the signals activating the ERK pathway are initiated through receptor-mediated activation of Ras [4] by stimulating the exchange of GDP bound to Ras for GTP [91]. Then, Ras phosphorylates Raf-1. Then, a MAPK cascade is initiated in which Raf-1 sequentially phosphorylates MEK1/2 and ERK1/2. Later, ERK1/2 translocate to the nucleus in a process that culminates in modulation of gene transcription through the activation of several transcription factors such as Ets-1 [4], ATF-2, c-Fos, c-Myc, Elk-1 [92], or NF-*κ*B [29] (Figure 1). At the same time, ERK1/2 can also phosphorylate cytoplasmic and nuclear kinases, such as MNK1, MNK2, MPKAP-2, RSK, or MSK1 [90].

TGF-*β* and EGF are growth factors that can induce tumor progression by means of the ERK pathway [93–96]. Several studies showed that these factors are overexpressed in prostate cancer in comparison with normal tissue [95–98]. In different tumor cells, expression of some EGF family members such as EGF or TGF-*α* is associated with poor patient prognosis or resistance to chemotherapeutics [94–99]. IGF-1 and EGF stimulate intracellular signaling pathways converging at the level of ERK2 [100], which is a key kinase mediator of growth-factor-induced mitogenesis in prostate cancer cells [101]. The two major substrates of the IGF-1 receptor, insulin receptor substrate-1 [102] and Shc, are known to contribute to IGF-1-induced activation of ERK [103].

The ERK signaling pathway plays a role in several steps of tumor development [14]. In fact, some components of the Raf-MEK-ERK pathway are activated in solid tumors (such as prostate or breast cancer) and hematological malignances [104–106]. In approximately 30% of human breast cancers, mutations are found in the ERK1/2 MAPK pathway [65]. ERK1/2 and downstream ERK1/2 targets are hyperphosphorylated in a large subset of mammary tumors [107]. Mutations of K-Ras appear frequently in many cancers including those of the lung and colon [108]. Mutations in the B-Raf gene are responsible for 66% of malignant melanomas [109]. Increased expressions of Raf pathway has been associated with advanced prostate cancer, hormonal independence, metastasis, and a poor prognosis [110]. Moreover, prostate cancer cell lines isolated from patients with advanced cancer (LNCaP, PC3, DU145) expressed low levels of active Raf kinase inhibitors [105]. TNF-*α* acts as an ERK activator in some cases related to inflammation and cell proliferation. In this way, Ricote et al. [11] showed that ERK phosphorylation was notably increased by TNF-*α* in a dose-dependent manner in LNCaP cells. In prostate cancer, presence of Raf-1 and MEK1 in conjunction with elevated ERK1 and ERK2, and their phosphorylated forms, suggests that stimulation of cell proliferation could be triggered by IL-6 via the ERK pathway [104]. In fact, IL-6 expression increased in prostate cancer in comparison with normal tissue [104, 111]. Moreover, LNCaP cells which produce IL-6 show increased proliferation, at least in part, due to ERK activation [112]. Recently, a phase I clinical trial has revealed the ability of an anti-IL-6 antibody (siltuximab) to inhibit ERK1/2 phosphorylation in prostate tumors [113]. 

Several investigators suggest associations between decline in ERK activity and advanced malignancy [114, 115]. Conversely Gioeli et al. [116] demonstrated that ERK activation is correlated with tumor malignancy. Junttila et al. [4] demonstrated in the TRAMP mouse model that ERK activation is linked to prostatic epithelial proliferation and initiation of prostate cancer development, while ERK inactivation is correlated with the emergence of a poorly differentiated metastatic and androgen-independent phenotype. Activated ERK mediates activation of the androgen receptor and/or PSA secretion through the growth factor receptor tyrosine kinase, Her2/Neu (also known as erbB2) in androgen-independent prostate cancer cells [117]. Other important issue of this pathway in tumor development is that the phosphorylation by ERK of proteins such as myosin, calpain, focal adhesion kinase, and paxillin promotes cancer cell migration. Also, ERK can promote the degradation of extracellular matrix proteins and consequent tumor invasion [14].

ERK may also induce the phosphorylation of apoptotic regulatory molecules including bcl-2 family members (e.g., Bad, Bim, and controversially Bcl-2) and caspase 9 [93]. There are pieces of evidence suggesting a protective effect in cells by NF-*κ*B activation via ERK [118, 119]. Upon cell stimulation NF-*κ*B is translocated into the nucleus [120], where it promotes the expression of several antiapoptotic genes such as inhibitors of apoptosis proteins (IAPs) [121] and bcl-2 family members [122].

## 5. ERK5

The fourth MAPK of interest in this paper is ERK5. ERK5 is a large molecular size kinase [123] identified independently by two groups. One used a two-hybrid screen with an upstream activator MEK5 as the bait; the other used a degenerate PCR strategy to clone novel MAPK [123, 124]. ERK5 is activated by growth factors [125], integrin engagement [126], and cell stress [111] and contributes to expression induction of Ap1 (cJun [127] and Fos [128]), MEF family group (e.g., MEF2C, a well-characterized target [129], and c-Myc [130] transcription factors).

In an *in vitro* study on androgen-independent PC3 cells, McCracken et al. [131] described ERK5 overexpresion related to proliferative, migrative, and invasive capabilities, establishing the potential importance of ERK5 in aggressive prostate cancer. In other study, Sawhney et al. [126] hypothesized that ERK5 activation could promote cancer metastasis through its ability to regulate cell adhesion and motility.

## 6. New Perspectives

The literature reviewed in this paper suggests that the MAPK transduction pathways are involved in prostate cancer development. The ability of JNK, p38, and ERK to act either as prostate cancer suppressors or promoters depends on the cell type, developmental stage, and specific stimuli. Nevertheless, the molecular roles of these proteins are not known at all. The aim of future studies might be directed towards revealing the factors and mechanisms that account for the differential function of JNK, p38, and ERK MAPKs as pro- or antitumor factors. It may lead to the development of therapeutic approaches to effectively target the protumor effects of the MAPK pathways.
